# IL-17 and IL-22 in Cerebrospinal Fluid and Plasma Are Elevated in Guillain-Barré Syndrome

**DOI:** 10.1155/2012/260473

**Published:** 2012-10-02

**Authors:** Shujuan Li, Ming Yu, Haifeng Li, Hongliang Zhang, Yanfang Jiang

**Affiliations:** ^1^Department of Neurology, The First Hospital, Jilin University, Jilin Province, Changchun 130021, China; ^2^Department of Neurology, The Affiliated Hospital of Jiangsu University, Jiangsu Province, Zhenjiang 212001, China; ^3^Department of Neurology, The Affiliated Hospital of Medical College, Qingdao University, Shandong Province, Qingdao 266003, China; ^4^Department of Central Laboratory, The Second Part of the First Hospital, Jilin University, Jilin Province, Changchun 130032, China

## Abstract

Guillain-Barré syndrome (GBS) is an acute autoimmune-mediated inflammatory demyelinating disease that causes rapidly progressing paralysis and occasionally respiratory failure. We hypothesized that interleukin (IL)-17 and IL-22 are elevated in GBS and participate in the autoimmune inflammatory response of GBS. We used sandwich enzyme-linked immunosorbent assay (ELISA) to measure the IL-17 and IL-22 levels in the CSF, and plasma from 22 GBS patients at the acute phase and 18 healthy controls (HC). The results show that CSF and plasma levels of IL-17 and IL-22 are elevated in GBS patients compared with HC. IL-17 and IL-22 levels in CSF, respectively, are correlated with GBS disability scale scores (GDSs). Meanwhile, IL-17 and IL-22 levels in CSF, IL-22 in CSF, and plasma of GBS patients have positive correlation, respectively. The increased levels of IL-17 and IL-22 in CSF may be explained by the disruption of blood-brain barrier (BBB) and peripheral nervous system (PNS) local inflammation in GBS. Meanwhile, the elevated levels of these two cytokines in plasma suggest the activation of Th17 and Th22 cells in the systemic immune response of GBS. Our data provide preliminary evidence that GBS is associated with high levels of IL-17 and IL-22 in CSF and plasma. These cytokines display pathogenic potential and may serve as useful biomarkers for GBS.

## 1. Introduction

Guillain-Barré syndrome (GBS) is an acute autoimmune-mediated inflammatory demyelinating disease that affects the peripheral nerves involving myelin sheath and axons [1]. It is clinically characterized by rapidly progressing symmetrical weakness and hyporeflexia/areflexia followed mostly by recovery [1, 2]. The severity of weakness ranges from mild limb asthenia to absolute paralysis, occasionally with respiratory failure that may lead to death [1, 2]. Meanwhile, the cerebrospinal fluid (CSF) of GBS patients usually shows characteristic albumin-cytological dissociation, that is, elevated protein levels and approximately normal cell counts, from two weeks after the disease onset [2]. There is strong evidence proving that both humoral and cellular immune mechanisms are involved in the pathogenesis of GBS: (i) serum antibodies to various gangliosides in human peripheral nerves have been found in about half of GBS patients [3]; (ii) pathological findings in GBS include lymphocytic infiltration in spinal roots and peripheral nerves, followed by macrophage-mediated, multifocal stripping of myelin [4]; (iii) plasma exchange (PE) and intravenous immunoglobulin (IVIg) therapies are effective in the treatment of GBS patients [5, 6]. However, the exact immunological mechanism of GBS remains not understood.

Interleukin (IL)-17 is mainly produced by T helper (Th) 17 cells, a recently identified lineage of CD4+ Th cells [7]. Both IL-17 and Th17 cells play a critical role in host defense responses and inflammatory diseases [7]. Besides IL-17, IL-22 is another Th17 effector cytokine that was originally termed as IL-10-related T-cell-derived inducible factor [8]. In experimental models of infectious diseases, IL-22 plays a crucial role in mucosal host defense in the lung and intestine by increasing epithelial cell proliferation and inducing anti-microbial peptides [9, 10]. IL-22 has been shown to mediate either detrimental or beneficial inflammatory responses in different conditions [11].

Studies showed that both IL-17 and IL-22 are functional cytokines in multiple autoimmune and inflammatory diseases. Matusevicius et al. found that multiple sclerosis (MS) patients have increased numbers of IL-17A mRNA-positive mononuclear cells in both the peripheral blood and the CSF [12]. Serum and synovial fluid levels of IL-17 were in correlation with disease activity in patients with rheumatoid arthritis (RA) [13]. IL-17 expression was also elevated in the serum of patients with inflammatory bowel disease (IBD) [14]. There was an increased level of IL-22 in serum of psoriasis [15] and Crohn's disease [16]. Moreover, the expression of IL-22 was also clearly associated with the severity of these diseases [15, 16]. Beyeen et al. confirmed that IL-22R *α*2 (*IL-22A2*) is an MS-risk gene through genetic and immunological investigation in the MS model experimental autoimmune encephalomyelitis (EAE) and large-scale association studies of MS patients [17].

Although IL-17 and IL-22 have been extensively studied in multiple inflammatory and autoimmune diseases, their correlation with GBS has not been reported yet. We hypothesized that IL-17 and IL-22 are related to GBS by means of participating in the focal and systemic immune response in GBS patients. So our study was performed to evaluate the CSF and plasma levels of these two cytokines at the acute phase of GBS, and to find out the correlations between these two cytokines and functional disability scales as well as various CSF parameters.

## 2. Materials and Methods

### 2.1. Patients

All subjects are from the Department of Neurology, the First Hospital, Jilin University, Changchun, or from the Department of Neurology, the Affiliated Hospital of Medical College, Qingdao University, Qingdao, China during September 2010 to March 2012. After informed consents were obtained, paired samples of plasma and CSF were collected from 22 GBS patients (8 females and 14 males, age 19–65 years) fulfilling international diagnostic criteria for GBS or its variants [18] and 18 healthy controls (HC). At the time of sampling, none of the patients had received any immunomodulatory drugs within the past three months. Patients with chronic immune-mediated disorders were excluded. Eighteen age- and sex-matched healthy donors (7 females and 11 males, age 21–57 years) were included as the control group. The protocols were approved by the Human Ethics Committee of Jilin Province and the Human Ethics Committee of Qingdao University, China.

### 2.2. Sample Collection

CSF and plasma samples were obtained during standard diagnostic lumbar puncture and peripheral vein puncture, respectively, during the first 2 weeks after the onset of GBS and stored at −80°C until use.

### 2.3. Evaluation of GBS Disease Severity

The severity of GBS was scored by the use of GBS disability scale scores (GDSs), a widely accepted scoring system to evaluate the functional status of GBS patients [19], that is, grade 0 = normal neurological status; grade 1 = minor symptoms, able to run; grade 2 = limb weakness, able to walk 5 m unaided; grade 3 = able to walk 5 m only with aid; grade 4 = chair or bed bound; grade 5 = requiring assisted ventilation; grade 6 = death. The correlation of various antibody parameters with disease severity was tested.

### 2.4. Cytokine Measurements Using Enzyme-Linked Immunosorbent Assay (ELISA)

The CSF and plasma IL-17 and IL-22 levels were detected by double antibody sandwich ELISA method. Mouse anti-human IL-17 and IL-22 monoclonal antibodies (mAb) as capture antibodies, biotinylated antibody reactive with human IL-17 and IL-22 as detecting antibodies, and recombinant human IL-17 and IL-22 proteins as standard controls, were used in this study (All from R&D systems, Minneapolis, USA). All procedures were performed according to the manufacturer's instructions. Briefly, flat bottom, high-binding capacity 96-well ELISA plates (Greiner Bio-One, Frickenhausen, Germany) were coated with 100 *μ*L IL-17 (4 *μ*g/mL) mAb and IL-22 (4 *μ*g/mL) mAb in carbonate bicarbonate buffer (PH 9.6) and kept at 4°C overnight. After several washes using phosphate-buffered saline (PBS) with 0.5% Tween-20 (PBST), the wells were blocked with 360 *μ*L 1% bovine serum albumin (BSA) (Sigma, St. Louis, MO, USA) per well for 2 hours (h) at room temperature (RT). After extensive washing with PBST, 100 *μ*L plasma and CSF samples diluted at 1 : 2 in PBS with 0.1% BSA, and recombinant standard proteins were added to each well for 2 h incubation at RT. Thereafter, the plates were washed with PBST, and 100 *μ*L biotinylated antibodies IL-17 (0.20 *μ*g/mL) and IL-22 (0.27 *μ*g/mL), respectively, were added to each wells. After 2 h incubation at RT and three washes with PBST, 100 *μ*L freshly prepared streptavidin-HRP (R&D systems) diluted at 1 : 200 in PBS with 0.1% BSA was added to each well for 1 h at RT. After three washes with PBST, 100 *μ*L enzyme substrate that is mixed with equal volumes of stabilized hydrogen peroxide and stabilized tetramethylbenzidine (both from R&D systems) was added to perform color reaction. Finally, after 20 min incubation in the dark, 100 *μ*L of sulfuric acid was added to stop the reaction. Optical density (OD) was determined at 450 nm by enzyme-labeled meter (Bio-Rad 680, Hercules, CA, USA). In order to quantify IL-17 and IL-22 in plasma and CSF, standard IL-17 and IL-22 curves were obtained simultaneously by incubating recombinant IL-17 at different known concentrations (0, 1.56, 3.13, 6.25, 12.50, 25, 50, and 100 pg/mL) and IL-22 (0, 31.25, 62.50, 125, 250, 500, 1000, and 2000 pg/mL). OD values measured from the standard concentrations of IL-17 and IL-22 were used to plot standard curves using computer software. The concentrations of proteins were quantified by extrapolation from the standard curve. All assays were done in duplicates.

### 2.5. Statistical Analysis

Data were expressed as the mean ± standard deviation (SD). For statistical analysis, differences of mean values were tested with the student's *t*-test for two groups, using SPSS 17.0 (SPSS, IBM, West Grove, USA). The Pearson or Spearman correlation coefficient was used to analyze correlations depending on data distribution. Reported *P*-values are two-tailed and considered statistically significant at *P* < 0.05.

## 3. Results

The demographic features of the studied subjects, their CSF data, and the individual CSF and plasma concentrations of IL-17 and IL-22 in GBS patients and HC of this study were displayed in Table 1.

### 3.1. CSF and Plasma Levels of IL-17 and IL-22 Are Elevated in GBS Patients

**Figure 1:**
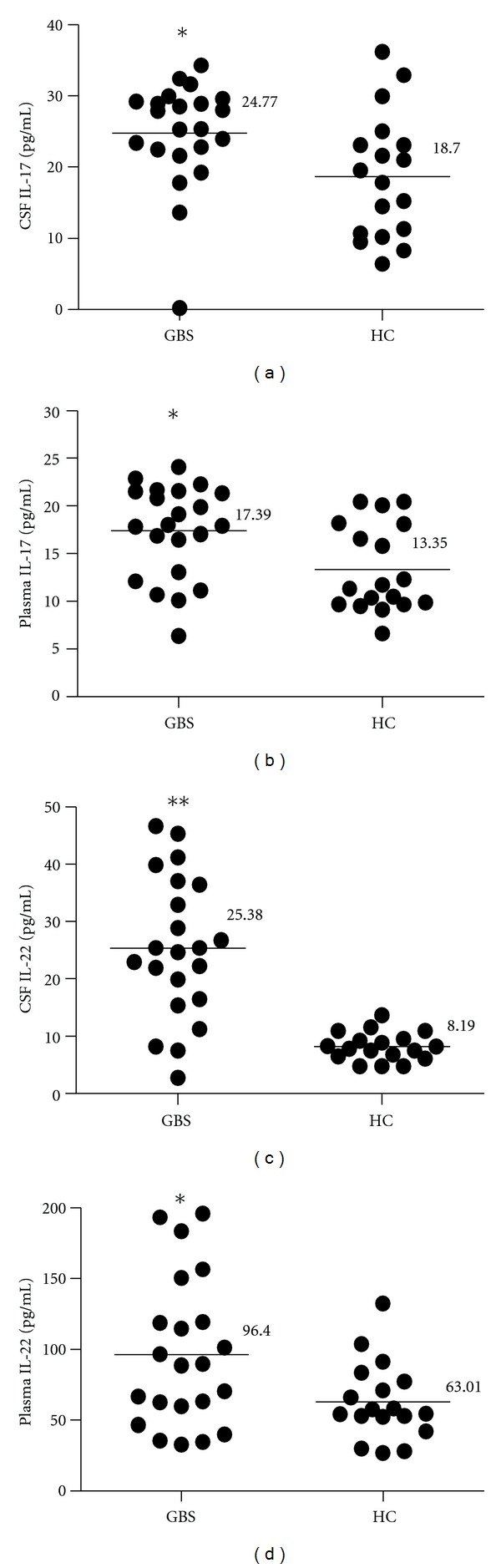
CSF and plasma levels of IL-17 and IL-22 in GBS (*n* = 22) and healthy controls (HC, *n* = 18). Statistical significance was indicated: *P < 0.05, **P < 0.001. Each circle = single individual, horizontal bars = mean.

The levels of IL-17 and IL-22 in both CSF and plasma were elevated in all GBS patients. The elevation was most obvious for IL-22 in CSF (*P* < 0.001), followed by IL-17 in CSF (*P* = 0.023), IL-17 in plasma (*P* = 0.011), and IL-22 in plasma (*P* = 0.019), when compared with HC. Meanwhile, the mean levels of IL-17 in CSF were higher than in plasma of both GBS patients and HC, whereas the mean levels of IL-22 in CSF were lower than in plasma in both groups (Figure 1; Table 1).

### 3.2. IL-17 and IL-22 Levels in CSF, Respectively, Are Correlated with GBS Severity

**Figure 2:**
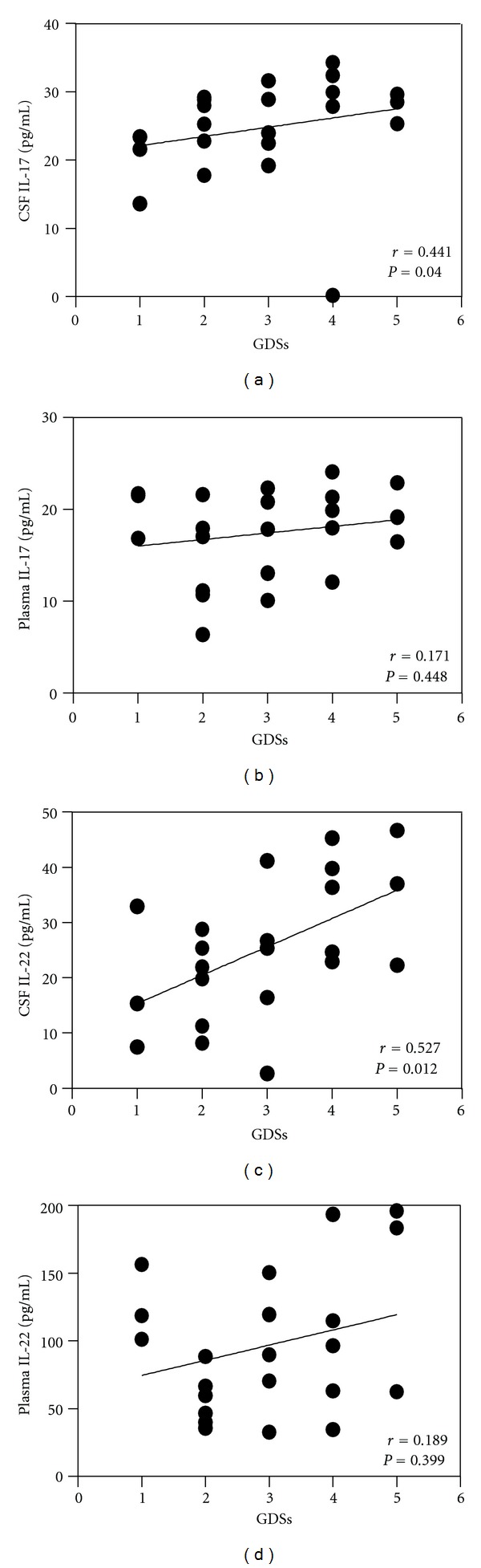
The correlations of CSF and plasma levels of IL-17 and IL-22 with GBS disability scale scores (GDSs). The Spearman correlation coefficient was used to analyze between two sets. Each circle = single individual; lines = linear approximation.

The Spearman correlation coefficient was used to analyze the correlation between cytokine levels and GDSs. IL-17 and IL-22 levels in CSF had positive correlation with GDSs (*r* = 0.441, *P* = 0.040; *r* = 0.527, *P* = 0.012, resp.). However, there was no statistical correlation between IL-17 and IL-22 in plasma and GDSs (*r* = 0.171, *P* = 0.448; *r* = 0.189, *P* = 0.399, resp.). Although there was no statistical significance, the elevated level of IL-22 in plasma had a tendency toward positive correlation with GBS severity (Figure 2).

### 3.3. The Level of IL-17 Is Correlated with That of IL-22 in CSF, and the Level of IL-22 in CSF Is Correlated with that in Plasma

**Figure 3:**
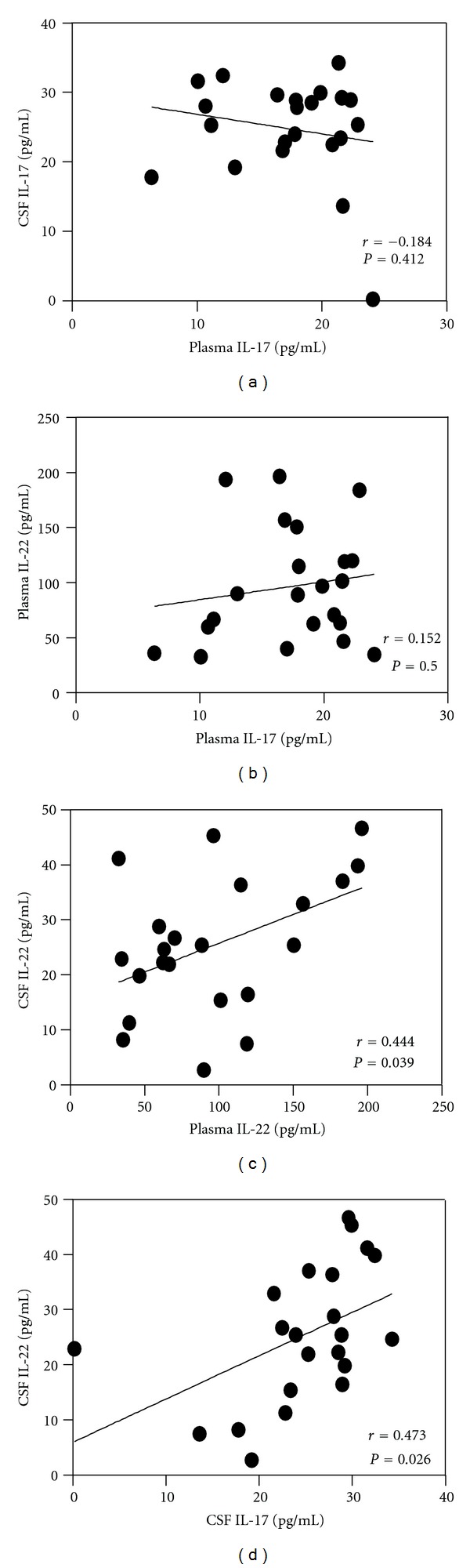
The correlations of CSF and plasma levels of IL-17 and IL-22. The Pearson correlation coefficient was used to analyze between two sets. Each circle = single individual; lines = linear approximation.

The Pearson correlation coefficient was used to analyze two parameters. The levels of IL-17 and IL-22 in CSF were positively correlated (*r* = 0.473, *P* = 0.026). So were the levels of IL-22 in CSF and plasma (*r* = 0.444, *P* = 0.039). However, no correlation was found between plasma IL-17 and CSF IL-17 levels (*r* = −0.184, *P* = 0.412), or between plasma IL-17 and plasma IL-22 levels (*r* = 0.152, *P* = 0.500) (Figure 3). However, neither the level of IL-17 nor that of IL-22 (in CSF and in plasma) had any correlation with the CSF data (including the white blood cell counts, the total protein concentration, and the albumin concentration) and Qalb in GBS patients (data not shown).

### 3.4. The Levels of Total Protein and Albumin in CSF, and the Albumin Quotient (Qalb) Values Are Increased in GBS

The total protein and albumin levels in CSF of GBS patients were increased compared with those of HC (*P* = 0.016 and *P* = 0.005, resp.). Qalb was defined as the ratio of CSF to plasma albumin concentrations, which was elevated in GBS compared with HC (*P* = 0.013). No statistical significance regarding the albumin level in plasma and white blood cells in CSF was found between GBS patients and HC (Table 1). 

## 4. Discussion

The IL-17 family includes structural related cytokines, of which IL-17A, IL-17B, IL-17C, IL-17D, IL-17E, and IL-17F are identified. These family members can induce the expression of proinflammatory cytokines such as tumor necrosis factor (TNF)-*α* and IL-1*β* to promote neutrophil migration, so they play inflammatory roles in the development of diseases [20]. IL-22 can be secreted by Th22 cells as well as Th17 cells, *γδ* T cells, and NK cells [11]. IL-22 has high similarities in the structure of encoding genes and protein with IL-10 [21]. In contrast to the anti-inflammatory properties of IL-10, however, IL-22 shows proinflammatory properties [22].

GBS is classically regarded as T helper (Th)1 cells-mediated autoimmune inflammatory disease. Whereas, some changes in experimental autoimmune neuritis (EAN), an animal model of human GBS could not be explained by this opinion perfectly. Zhang et al. found that IFN-*γ* knockout mice were also susceptible to EAN [23], this indicated other factors and mechanisms are involved. Moreover, IL-17A positive cells in cauda equina (CE) infiltrating cells, and the levels of IL-17A in sera were increased in IFN-*γ* knockout mice [23]. IL-17 was found in sciatic nerves of EAN, and the accumulation of IL-17 was correlated with the severity of neurological signs [24], which suggested a pathological contribution of IL-17 to the development of EAN. The frequency of Th17 cells in CSF and the level of IL-17 in plasma were significantly higher in active chronic inflammatory demyelinating polyradiculoneuropathy (CIDP) [25]. There was strong evidence for proving that IL-17 had some correlation with GBS.

 Our data showed that levels of IL-17 and IL-22 in CSF and plasma of GBS patients were obviously elevated compared with HC. This is the first human study reporting an elevation of IL-17 and IL-22 levels in GBS, although the functional roles of IL-17 and IL-22 in GBS require further investigation. In previous studies, IL-6 in the presence of transforming growth factor (TGF)-*β*1, and IL-6, IL-23 in combination with IL-1*β* had been proved to be the effective factors responsible for the differentiation of Th17 cells and the production of IL-17; these cytokine milieux may also facilitate the expression of IL-22 [26, 27]. IL-6, IL-1*β*, IL-23, and TNF-*α* are all proinflammatory cytokines and have an important role in the pathogenesis of GBS [28]. Therefore, it is safe to conclude in our study that the extensive network of IL-17 and IL-22 in coordination with these inflammatory cytokines is associated with the pathogenesis of GBS. The elevated levels of IL-17 and IL-22 in plasma reflect the activation of Th17 and Th22 cells in the systemic auto-immune responses of GBS patients. Therefore, IL-17 and IL-22 might be important effectors in addition to Th1 cells and Th1 cytokines in autoimmune-mediated responses in GBS. The male-to-female ratio in our study was 1.75 : 1, which was in line with some previous reports [29], and this confirmed that GBS might be a kind of male-vulnerable autoimmune disease. We presume that estrogen and testosterone may influence the production of inflammatory cytokines, such as IL-17 and IL-22. But the exact mechanisms are still unknown and need further researches.

Our data showed that GBS patients had increased IL-17 and IL-22 concentrations in CSF. In addition, in line with previous studies, Qalb, the total protein and albumin level of CSF were remarkably higher in GBS patients [30]. CSF is considered to reflect events within the blood-nerve barrier (BNB), and it is more approximate to target organs of GBS than the peripheral blood. Kieseier reported that immune-mediated disorders of the peripheral nervous system (PNS) are pathologically characterized by the breakdown of the BNB, accumulation of activated T cells and macrophages in the PNS, and demyelination of peripheral nerves [31]. Human Th17 cells could disrupt the tight junctions of blood-brain barrier (BBB) by direct effects of IL-17A and IL-22 on endothelial cells [32]. GBS is a reported autoimmune demyelination disease affecting both the central nervous system (CNS) and PNS [33], meanwhile, our data of the elevated IL-17 and IL-22 levels in CSF and plasma of GBS confirmed this respect. We presume several potential mechanisms of the origin in CSF and plasma of these cytokines: (i) IL-17 and IL-22 in peripheral blood could penetrate the BBB, so the levels of them in CSF are also elevated; (ii) the elevation of IL-17 and IL-22 in CSF and plasma might be related to the local inflammation of spinal roots and peripheral nerves that causes demyelination and axon degeneration; (iii) reactive Th17 and Th22 cells in peripheral blood may cross the BBB/BNB and secret relative cytokines. 

We also found that the CSF levels of IL-17 and IL-22, respectively, were correlated with GDSs at the acute phase of GBS, and there was a positive correlation between them. Nevertheless, the levels of IL-22 in CSF and plasma also had a positive correlation. This indicates that the elevation of IL-17 and IL-22 in CSF is related to the severity of inflammation in the spinal roots. Since their elevations in CSF are synchronous, IL-17 and IL-22 may coordinate in the pathogenesis of the disease and may be a biomarker for indicating disease severity or prognosis, which, however, should be confirmed in future studies with a larger sample size.

There are limitations in our study. Due to the low incidence of GBS in the whole population [3], the sample size is relatively small. Although paired samples of CSF and plasma were collected, the time-points of lumbar puncture with regard to disease onset varied among GBS patients. These limitations may influence the statistical power of the data analysis.

In summary, this study showed increased CSF and plasma levels of IL-17 and IL-22 in GBS patients compared with HC. We also demonstrated that the levels of IL-17 and IL-22 in CSF are correlated with GBS severity. Nevertheless, the level of IL-17 was correlated with that of IL-22 in CSF. Meanwhile, the levels of IL-22 in CSF and plasma also had a positive correlation. Further studies are warranted to confirm the immunological mechanisms of the elevated IL-17 and IL-22 levels in GBS.

## Figures and Tables

**Table 1:** Demographic data and IL-17 and IL-22 levels in the cerebrospinal fluid (CSF) and plasma of Guillain-Barré syndrome (GBS) patients and healthy controls (HC).

	GBS (*n* = 22)	HC (*n* = 18)
Female/male	8/14	7/11
Age	41.91 ± 13.16	39.11 ± 11.18
White blood cells in CSF (per *μ*L)	1.32 ± 1.21	1.06 ± 0.94
Total protein level in CSF (mg/dL)	71.56 ± 30.79*	52.01 ± 12.81
Albumin level in CSF (mg/dL)	49.58 ± 25.33**	30.27 ± 11.71
Albumin level in plasma (g/L)	41.55 ± 12.56	43.67 ± 14.66
Qalb × 100	1.24 ± 0.60*	0.79 ± 0.45
IL-17 concentration in CSF (pg/mL)	24.77 ± 7.45*	18.70 ± 8.72
IL-17 level in plasma (pg/mL)	17.39 ± 4.87*	13.35 ± 4.54
IL-22 concentration in CSF (pg/mL)	25.38 ± 12.35***	8.19 ± 2.50
IL-22 level in plasma (pg/mL)	96.40 ± 52.28*	63.01 ± 27.22

Data are expressed as mean ± SD, student's *t*-test. *: Statistically significant in comparison with HC, *P < 0.05, **P < 0.01, ***P < 0.001.

Qalb = CSF albumin/plasma albumin.
